# Therapeutic storywriting in the context of eating disorders: working around resistance in order to facilitate insight and explore possibilities for change

**DOI:** 10.1186/2050-2974-3-S1-P14

**Published:** 2015-11-23

**Authors:** Claudia Foltun

**Affiliations:** 1 Thrive - Eating Disorders Treatment, Auckland, New Zealand

## 

Therapeutic storywriting has been shown to reduce behavioural, emotional and social difficulties by providing a non-threatening way to explore and process difficult situations. Clients undergoing treatment for Anorexia Nervosa (AN) were introduced to working with metaphors and were encouraged to create a symbolic character and a story that reflects their experience with the eating disorder (ED). By working through the emotional safety of story metaphors, the clients were able to identify and discuss issues that might otherwise be overwhelming or shameful to share. Clients expressed in images and symbols the evolution of their ED, explored its impact on current functioning, and generated metaphoric solutions for change. Several examples of such client story metaphors are provided, along with solutions generated by the client or the therapy group.

